# Pool walking may improve renal function by suppressing the renin-angiotensin-aldosterone system in healthy pregnant women

**DOI:** 10.1038/s41598-020-59598-9

**Published:** 2020-02-19

**Authors:** Tatsuya Yoshihara, Masayoshi Zaitsu, Shiro Kubota, Hisatomi Arima, Toshiyuki Sasaguri

**Affiliations:** 1 Department of Clinical Pharmacology, Faculty of Medical Sciences, Kyushu University, Maidashi 3-1-1, Higashi-ku, Fukuoka, 812-8582 Japan; 2 Clinical Research Center, Fukuoka Mirai Hospital, Kashiiteriha 3-5-1, Higashi-ku, Fukuoka, 813-0017 Japan; 3 Department of Social and Behavioral Sciences, Harvard T.H. Chan School of Public Health, 677 Huntington Avenue, 7th Floor, Boston, Massachusetts 02115 USA; 4 Department of Public Health, Graduate School of Medicine, The University of Tokyo, 7-3-1 Hongo, Bunkyo-ku, Tokyo, 113-0033 Japan; 5 Kubota Maternity Clinic, Fukuoka, Japan; 6 Kubota Life Science Laboratory Co., Ltd., Saga, Japan; 7 Department of Preventive Medicine and Public Health, Faculty of Medicine, Fukuoka University, Nanakuma 8-19-1, Jonan-ku, Fukuoka, 814-0180 Japan

**Keywords:** Cardiology, Disease prevention, Kidney

## Abstract

This study aimed to examine the effect of pool walking on renal function in pregnant women. Fifteen pregnant women (mean gestational age, 37.8 weeks) walked in a pool (depth 1.3 m) for 1 h. A few days later, they walked on a street for 1 h. Within each activity, the starting and ending levels of plasma renin activity were measured. The total urine volume, creatinine clearance, and change in plasma renin activity levels between each activity were compared by Wilcoxon rank-sum test. The renin-angiotensin-aldosterone level was suppressed during pool walking: the mean starting and ending values of plasma renin activity and serum aldosterone were 6.8 vs. 5.5 ng/mL/h (p = 0.002) and 654 vs. 473 pg/mL (p = 0.01), respectively. The decreases in plasma renin activity and serum aldosterone levels were more evident in pool walking than in land walking (plasma renin activity, −1.27 vs. 0.81 ng/mL/h, p = 0.002; serum aldosterone, −180.9 vs. 3.1 ng/mL/h, p = 0.03), resulting in higher total urine volume (299 vs. 80 mL, p < 0.001) and creatinine clearance (161.4 vs. 123.4 mL/min, p = 0.03) in pool walking. Pool walking may improve renal function in pregnant women partly through the suppressed renin-angiotensin-aldosterone system.

## Introduction

Preeclampsia, along with other hypertensive disorders of pregnancy, is one of the major contributors for maternal mortality^1–4^. Currently, up to 10% of pregnant women have hypertensive disorders of pregnancy^5^, and the prevalence has been increasing over time particularly in (but not limited to) developed countries^2,3^. Although pharmaceutical (e.g., antiplatelet agents) and dietary approaches may potentially prevent preeclampsia^6–8^, advantages of behavioral interventions (e.g., maternal physical activities) are still unclear^9^.

Although the biological pathogenesis of preeclampsia has not been fully identified, it is recognized that preeclampsia is not only a disease of high blood pressure/renal dysfunction but also a systemic disease associated with placental hypoperfusion/hypoxia with oxidative stress, inflammation, and endothelial/angiogenesis modifiers^1,2,5,10^. Studies also suggest that other endocrine systems, for example, vasopressin, are involved in the pathogenesis of preeclampsia^11^. Interestingly, a high salt intake lowered blood pressure in pregnant women and animals^12,13^. According to several physiological studies for aquatic activities, hydrostatic pressure appeared to improve circulatory perfusion and suppress the renin-angiotensin-aldosterone system^14^, and some studies addressed aquatic physical activities during pregnancy^15–17^. However, few studies have *specifically* assessed the association between aquatic physical activities during pregnancy and downregulation of the renin-angiotensin-aldosterone system^18^. We hypothesized that hydrostatic pressure mobilizes peripheral fluid (edema) to the intravascular space, leading to transient volume expansion, downregulation of the renin-angiotensin-aldosterone system, and diuresis in pregnant women.

Accordingly, the present study aimed to examine the effect of head-out pool walking on renal function in pregnant women. Using clinical and renal function data, we determined whether pool walking improved renal function (urine production and creatinine clearance) in combination with suppressed renin-angiotensin-aldosterone system.

## Methods

### Study settings

The trial was approved by the Kyushu University Hospital Clinical Research Ethics Board (approval number 24045) and registered in the University Hospital Medical Information Network (UMIN) Clinical Trials Registry (UMIN000009051 released on October 5, 2012). Written informed consent was obtained from all patients. All procedures performed in this study involving human participants were in accordance with the ethical standards of the institutional research committee and with the 1964 Declaration of Helsinki and its later amendments or comparable ethical standards.

An indoor swimming facility of the Fukuoka Swimming Club located in Fukuoka, Japan, was used for the prenatal aquatic activity of pool walking. Aquatic activity instructors, accredited for cardiopulmonary resuscitation, overviewed and led the pool walking activity. The Kubota Maternity Clinic in Fukuoka, Japan, a private, general obstetric and gynecological hospital that closed in 2017^19,20^, conducted the pool walking program along with the Fukuoka Swimming Club. Pool walking is common in Japan as an aquatic exercise, and indoor pool facilities usually provide a “walking” lane.

### Study design and participants

In the trial, participants walked in the indoor pool with the head-out position for 1 hour at their own pace. The dimensions of the pool having six lanes were as follows: length, 25 m (27.3 yds), and depth, 1.3 m (4.3 ft). The water temperature was set at 31–32 °C and the ambient room temperature at 29–30 °C. A few days later (2–9 days), the same participants walked on a street, that is, a conventional prenatal activity, near the Kubota Maternity Clinic for 1 hour at their own pace. We first planned to conduct this study in the typical two-arm crossover design by randomizing half of the participants to the reverse sequence. However, due to a potential risk based on our empirical experience that land walking induced labor occasionally, we finally decided not to choose the typical two-arm crossover design to prioritize the safety of our participants and their babies. Therefore, every participant firstly performed pool walking and subsequently land walking in the one-arm design. The weather conditions during land walking were fine/cloudy, the temperature was ~23–26 °C, and the humidity was ~ 40%–50%. During each activity, the participants were allowed to drink clear water freely (sodium compound <0.001 gram per 100 mL). Dates for each activity were determined according to the schedule of the participants.

The eligible participants in this trial were pregnant women (aged 20 to 39 years) with a normal pregnancy (gestational age, 37 weeks and above) and who had prenatal care at the Kubota Maternity Clinic and had already participated in the pool walking program before the study period. Participants were ineligible if they presented with cardiovascular disease, diabetes, chronic hypertension, kidney disease, hypertensive disorders of pregnancy, placenta previa/low-lying placenta, amniorrhexis, or anemia (hemoglobin <10 g/dl) or were considered not suitable for this study by physicians. We initially recruited 16 participants (11 participants in October 2012 and five participants in October 2013). We chose the trial date in October due to the general weather condition in Japan. One participant (37 years old at 38.4 weeks of gestation and who participated in 35 sessions of pool walking before the study) was excluded because she gave birth before performing the land walking exercise; therefore, only 15 participants were included in the analysis. Since there were no previous studies assessing the effect of pool walking in pregnant women, sample size was empirically determined by the sample size used in a previous study of water immersion^18^.

### Assessments of clinical and renal function parameters

In the trial, body weight, systolic and diastolic blood pressures, and pulse rate were measured at the start and end of each walking activity. Blood pressure was measured once at each measuring point in a relaxed, sitting position using an automatic noninvasive blood pressure monitor (HEM-7200, Omron, Kyoto Japan) with a standard size cuff (HEM-CR24 for upper arm circumference between 22 and 32 cm). Blood samples were collected at the start and end of each walking activity, and the starting and ending levels of plasma renin activity (PRA), serum aldosterone (SA), hematocrit, and serum creatinine were measured^18,21^. The serum osmolality was also assessed with serum sodium and potassium concentrations (2 × Na + 2 × K). Participants were required to remain seated <5 minutes. Within each walking activity, we calculated the changes in each parameter, subtracting the starting levels from the ending levels. According to the previous studies, a change in PRA was observed if the activity of the renin-angiotensin-aldosterone system was altered by excercise^18,21^.

Before each walking activity, all participants urinated. During each walking activity, all urine samples were collected, and the total amount of urine was recorded. With the total urine sample at the end of each walking activity, urine creatinine was measured. All laboratory measurements were performed at the Clinical Laboratory Center of Fukuoka City Medical Association, Fukuoka, Japan. We calculated creatinine clearance as follows: creatinine clearance = urine creatinine (mg/dL) × urine volume (mL/min)/([serum creatinine at the start + serum creatinine at the end]/2 [mg/dL]). The 1-hour creatinine clearance was comparable to the 24-hour creatinine clearance^22^.

### Statistical analysis

Between each activity, the starting levels of clinical and renal function parameters and the changes in these parameters were compared by Wilcoxon rank-sum test. The total urine volume and creatinine clearance were also compared by Wilcoxon rank-sum test. Within each activity, the starting and ending levels of each parameter were compared by Wilcoxon signed-rank test. Analyses for total urine volume and creatinine clearance were restricted to 14 participants since it was not possible to collect a sample from one participant during land walking.

Alpha value was set at 0.05, and all p-values were two-sided. Data were analyzed using JMP pro 12 (Statistical Analysis System Institute, Cary, NC, USA) and Stata/MP 13.1 (StataCorp, College Station, TX, USA).

## Results

The baseline characteristics of the participants are shown in Table 1. The starting levels of each parameter did not differ between pool and land walking activities (Table 1). The mean amount of water intake did not differ between activities (mean ± standard deviation: 179 ± 76 vs. 185 ± 56 mL, p = 0.92). The estimated amount of salt intake was small during the activities.

**Table 1:** Baseline characteristics of the study participants.

Characteristics	Mean (SD)		p*
	Before pool walking (n = 15)	Before land walking (n = 15)	
Age, y	32 (4)	—	—
Height, cm	160.1 (5.4)	—	—
Body weight before pregnancy, kg	51.8 (6.5)	—	—
Body mass index before pregnancy, kg/m^2^	20.2 (2.3)	—	—
Prior attendance to walking in water, hour	25 (13)	—	—
Gestational age, week	37.8 (1.0)	38.2 (1.0)	0.13
Body weight, kg	60.4 (6.8)	60.2 (6.7)	0.85
Body mass index, kg/m^2^	23.5 (2.2)	23.5 (2.1)	0.90
Systolic blood pressure, mmHg	115 (12)	111 (13)	0.51
Diastolic blood pressure, mmHg	69 (7)	68 (9)	>0.99
Pulse rate, beat per minute	81 (11)	79 (14)	0.66
Hematocrit, %	35.2 (2.1)	34.9 (2.0)	0.65
Plasma renin activity, ng/mL/h	6.8 (3.4)	6.3 (4.0)	0.44
Serum aldosterone, pg/mL	654 (518)	674 (407)	0.72
Serum creatinine, mg/dL	0.63 (0.08)	0.63 (0.06)	0.72
Serum osmolality, mOsm/kg H_2_O	281.5 (5.7)	282.1 (6.3)	0.52

*Wilcoxon rank-sum test.

**Figure 1:**
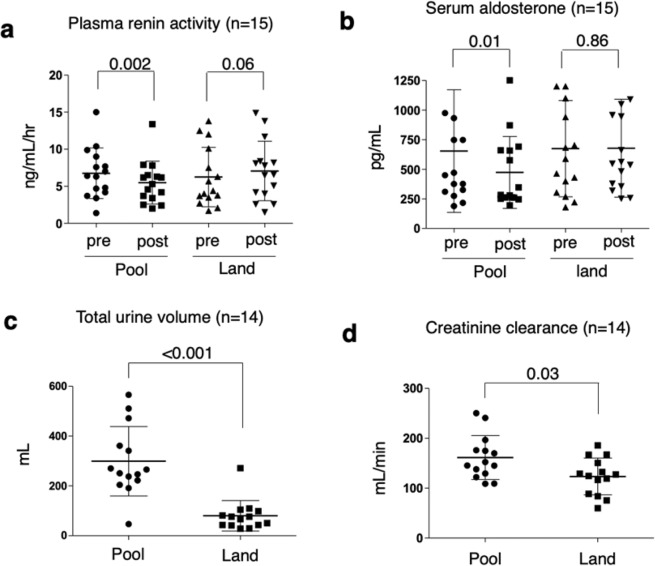
Renal function parameters during 1-h pool and land walking activities in 15 pregnant women. Differences between plasma renin activity levels and serum aldosterone levels before and after each activity (**a,b**). Total urine volume and creatinine clearance measured after 1-h pool and land walking activities (**c,d**). For total urine volume and creatinine clearance, data were available for 14 participants. Statistical analyses were as follows: (**a,b**) Wilcoxon signed-rank test and (**c,d**) Wilcoxon rank-sum test. The bars show the means and standard deviations.

Within each activity, the measurements were obtained before and at the end of 1 hour. These data are shown in Table 2. After pool walking, body weight, systolic and diastolic blood pressures, and hematocrit were not changed compared to levels measured before walking began. However, after pool walking, pulse rate, PRA, and SA significantly decreased (Table 2 and Fig. 1). Thus, PRA decreased from 6.8 ± 3.4 to 5.5 ± 2.9 ng/mL/h (p = 0.002), and SA decreased from 654 ± 518 to 473 ± 304 pg/mL (p = 0.01). By contrast, land walking had no effect on any of the parameters, although decreased body weight and pulse rate and increased PRA were possible (Table 2).

**Table 2:** Starting and ending levels of clinical and renal function parameters within each activity.

Characteristics	Mean (SD)					
	Pool walking (n = 15)			Land walking (n = 15)		
	Before	After	p^*^	Before	After	p^*^
***Starting and ending levels***						
Body weight, kg	60.4 (6.8)	60.3 (6.8)	0.46	60.2 (6.7)	60.1 (6.6)	0.09
Systolic blood pressure, mmHg	115 (12)	113 (12)	0.65	111 (13)	110 (9)	0.51
Diastolic blood pressure, mmHg	69 (7)	68 (7)	0.86	68 (9)	67 (5)	0.38
Pulse rate, beat/min	81 (11)	75 (11)	0.002	79 (14)	76 (12)	0.09
Hematocrit, %	35.2 (2.1)	35.4 (2.9)	0.49	34.9 (2.0)	35.0 (1.9)	0.82
Plasma renin activity, ng/mL/h	6.8 (3.4)	5.5 (2.9)	0.002	6.3 (4.0)	7.1 (4.0)	0.06
Serum aldosterone, pg/mL	654 (518)	473 (304)	0.01	674 (407)	677 (414)	0.86
Serum creatinine, mg/dL	0.63 (0.08)	0.63 (0.07)	0.91	0.63 (0.06)	0.64 (0.07)	0.18
Serum osmolality, mOsm/kg H_2_O	281.5 (5.7)	282.0 (3.2)	0.24	282.1 (6.3)	280.5 (3.0)	0.21
***Difference during each activity***	Pool walking (n = 15)			Land walking (n = 15)		p^†^
Body weight, kg	−0.05 (0.22)			−0.11 (0.21)		0.43
Systolic blood pressure, mmHg	−2.0 (9.8)			−1.8 (8.9)		>0.99
Diastolic blood pressure, mmHg	−0.3 (6.7)			−1.7 (8.3)		0.39
Pulse rate, beat/min	−6.4 (5.7)			−3.5 (7.2)		0.26
Hematocrit, %	0.26 (1.20)			0.15 (1.10)		0.92
Plasma renin activity, ng/mL/h	−1.27 (1.29)			0.81 (2.22)		0.002
Serum aldosterone, pg/mL	−180.9 (272.3)			3.1 (226.4)		0.03
Serum creatinine, mg/dL	0.00 (0.03)			0.01 (0.03)		0.28
Serum osmolality, mOsm/kg H_2_O	0.53 (5.04)			−1.67 (5.29)		0.11
***Renal function***	Pool walking (n = 14)			Land walking (n = 14)		p^†^
Total urine volume, mL	299 (139)			80 (61)		<0.001
Total creatinine excretion, mg	61 (15)			47 (15)		0.03
Creatinine clearance, mL/min	161.4 (44.0)			123.4 (36.9)		0.03

^*^Wilcoxon signed-rank test.

^†^Wilcoxon rank-sum test.

The results for the between-activity comparisons are shown in Table 2. Any changes in body weight, systolic and diastolic blood pressures, pulse rate, and hematocrit were not different between pool and land walking. However, the change in PRA and SA significantly differed between pool and land walking activities (Table 2): PRA, −1.27 ± 1.20 vs. 0.81 ± 2.22 ng/mL/h (p = 0.002), and SA, −180.9 ± 272.3 vs. 3.1 ± 226.4 ng/mL/h (p = 0.03). The total urine volume and creatinine clearance were significantly higher in pool walking than in land walking (Table 2 and Fig. 1). The mean total urine volumes after pool and land walking activities were 299 ± 139 and 80 ± 61 mL, respectively (p < 0.001). The mean creatinine clearances after pool and land walking activities were 161.4 ± 44.0 and 123.4 ± 36.9 mL/min, respectively (p = 0.03).

The baseline levels of serum osmolality did not differ between pool and land activities (Table 1), and it did not change during either activity (Table 2).

## Discussion

### Main findings

As hypothesized, we found that pool walking had lower PRA and SA levels compared to the conventional prenatal activity of land walking, and urine production and creatinine clearance increased. Although our results are based only on the effects of immersion and exercise on the renin-angiotensin-aldosterone system and further investigation of other pathways (e.g., vasopressin and salt intake) is required^11–13^, our findings support the previous findings demonstrating that head-out aquatic activities have potentially beneficial effects by improving central blood volume and perfusion of the kidneys and conceptus^14,18^.

### Interpretation

Hydrostatic pressure is probably responsible for the beneficial effects of pool walking activity. Hydrostatic pressure may mobilize peripheral fluid to the intravascular space, particularly from the expanded extracellular space of the lower extremities (edema), causing transient volume expansion, downregulation of the renin-angiotensin-aldosterone system, and diuresis in pregnant women^14,18,23,24^. Additionally, the renin-angiotensin-aldosterone system and sympathetic activity appeared to be suppressed in head-out water immersion with improved systemic/renal perfusion^14,24,25^. In our study, we observed an increase in urine volume and creatinine clearance, decreased renin-angiotensin-aldosterone levels, and decreased pulse rate during pool walking, suggesting an improved circulatory perfusion.

Another benefit of pool walking might be the added buoyancy for pregnant women. On land, the enlarged pregnant uterus might compress the inferior vena cava and abdominal veins through the force of gravity, leading to decreased cardiac preload/output^26,27^. By contrast, buoyancy in water might release the gravity-induced compression and improve cardiac preload/output^14^. Conventional physical activities such as land walking also have some benefits^1,5,15^, but strenuous exercise on land has the potential risks of reducing renal perfusion and urinary volume through vasoconstriction^14^.

### Strengths and limitations

The strengths of our study include a unique feature of light-intensity physical activity with the head-out pool walking, with vertical posture in water. This prenatal activity had the following advantages: it improved circulatory perfusion by hydrostatic pressure and did not require special skills (swimming/breathing techniques). Pool walking is also as safe as swimming in a pool regarding infectious risks. In our empirical experience, pregnant women felt more comfortable performing pool walking than swimming since they preferred pool walking (the attendance rates for swimming and pool walking activities were ~20% and ~80%, respectively).

Our study has some limitations. First, due to the limitation of the study, our study participants might be more health conscious compared with the general population of pregnant women, thereby limiting generalizability. Additionally, we could not assess the effect of pool walking in the reverse sequence (i.e., participant performs land walking first followed by pool walking). This one-arm design might potentially carry over the effect of pool walking to land walking. However, our participants walked on land at least 2 days after pool walking, and we confirmed that the baseline characteristics did not differ between the two activities. Additionally, previous studies stated that PRA levels return to baseline levels within a few hours after aquatic activities^28^. Therefore, it is unlikely that this limitation affects our findings. Second, in addition to our small sample size, we only assessed the renin-angiotensin-aldosterone system. Other parameters (circulatory and endocrine parameters, e.g., arterial diameters and arterial blood flow, catecholamines, atrial natriuretic peptide, and vasopressin) were not measured^11,18,27,29^. Third, while our trial demonstrated transient changes in the renin-angiotensin-aldosterone levels and renal function, questions remain about the value of pool walking in protecting against hypertensive disorders in pregnancy in the long-term and dose-response effects. We are currently gathering perinatal clinical data to clarify the long-term effect of pool walking in a cohort design, and these results will be incorporated in a future study. Additionally, further studies with a randomized controlled design are required to investigate the potential benefits of pool walking on cardiovascular health not only in pregnant women but also in patients with noncommunicable diseases such as diabetes and obesity^13^.

Finally, vasopressin is a potent vasoconstrictor^11^, and a decrease in vasopressin levels during pool walking is probably observed because any changes in central blood volume and inhibition of low-pressure baroreceptors would have had the same effect on vasopressin as that observed in PRA level in the current study. However, serum osmolality did not change during both pool and land walking activities, suggesting that the combination method of light physical exercise (walking) with hydrostatic pressure (water immersion) may not suddenly alter water-electrolyte balance but may rather improve circulatory perfusion by causing a gradual increase in plasma volume by the transcapillary autotransfusion of fluid. In this perspective, pool walking would be physiologically safe and beneficial in pregnant women’s cardiovascular health. We should further attempt to integrate all potential pathways related to circulatory perfusion, including the renin-angiotensin-aldosterone system, serum/urine osmolality, vasopressin and other relevant indicators (e.g., vasopressinase, copeptin, oxytocinase, and leucyl-cystinyl aminopeptidase), and behavioral factors (e.g., salt intake)^11–13,28–31^ to prevent preeclampsia.

## Conclusion

Pool walking activities may improve renal function in the short-term by suppressing the renin-angiotensin-aldosterone system in pregnant women, and potential long-term effects of pool walking will be evaluated in an ongoing study. Improved circulatory perfusion by hydrostatic pressure may be a promising indication for the prevention of preeclampsia, and aquatic facilities are available at community level (e.g., public indoor pools and swimming pools in school, hotel, and fitness club). Hence, public health and healthcare professionals, researchers, and pregnant women should focus further on the benefits of aquatic physical activities with hydrostatic pressure.
